# Thunderclap headache as an initial manifestation of acute aortic dissection: a case report and review of literature

**DOI:** 10.3389/fcvm.2025.1598757

**Published:** 2025-10-23

**Authors:** Mario Ranjeevan, Valeriia Nielsen, Egon Stenager, Sepehr Mamoei

**Affiliations:** ^1^ Department of Neurology, University Hospital of Southern Denmark, Aabenraa, Denmark; ^2^ Neurological Research Unit, University Hospital of Southern Denmark, Aabenraa, Denmark; ^3^ Department of Regional Health Research, University of Southern Denmark, Odense, Denmark

**Keywords:** headache, aortic dissection, computed tomography, neurologic deficits, emergency medicine, differential diagnosis

## Abstract

The authors describe the case of a 54-year-old man with sudden onset severe headache accompanied by transitory sensory-motor deficits in all extremities, and mild abdominal pain as the initial manifestation of acute aortic dissection. Despite clinical symptoms mimicking subarachnoid hemorrhage and transitory cerebral ischemia, CT-angiography of the cerebrum and thorax revealed a Stanford type A aortic dissection. Consequently, the patient underwent insertion of a prosthesis at the aortic root and arch. In this case report, we suggest a high level of suspicion for aortic dissection in patients presenting with a sudden severe headache and/or neurological deficits accompanied by chest/back/abdominal pain, nausea, and loss of consciousness. At the same time, asymmetry of pulse and blood pressure asymmetry of the arms should also raise suspicion. An acute CT angiography with the aim of timely diagnosis will allow earlier treatment for this life-threatening condition.

## Introduction

Acute aortic dissection (AAD) is a serious life-threatening condition often resulting in death due to cardiac tamponade and aortic rupture (1, 2). Clinical manifestations of aortic dissection are highly variable, which can challenge the diagnosis (2, 3). Untreated, it has a mortality rate of 50% in the first 3 days and 80% during the first two weeks (4). It usually manifests as a sudden excruciating pain anterior in the chest, which can radiate to the back (5). 17%–40% of all cases of aortic dissection present with neurological symptoms despite being a rare cause of ischemic stroke (6, 7). This is especially important for neurologists, as patients with focal neurologic symptoms due to acute aortic dissections who receive thrombolysis with recombinant tissue plasminogen activator (r-TPA) have a 71% mortality rate (7, 8). Furthermore, it has been estimated that aortic dissections are unsuspected in approximately 15%-43% of cases upon admission to hospitals, which underlines the importance of a high level of suspicion in a clinical setting (7, 9).

In this study, we present the case of a 54-year-old man who presented with thunderclap headache and focal neurological deficits as the presenting symptoms of acute aortic dissection.

## Case report

A 54-year-old man with no prior medical history, no use of medication, non-smoker, and no prior traumatic injuries in the days leading up to admission presented to the emergency department with a sudden thunderclap headache that originated in the neck and radiated to the top of the head. Within seconds, the headache became holocranial and was rated as 10 out of 10 on a numeric rating scale (NRS). This was followed by a sensation of pins and needles in his right arm, which spread to both legs, followed by weakness in both legs and his left arm. After a few minutes, the sensory-motor deficits on the right-sided extremities subsided, while symptoms on the left-sided extremities subsided after approximately 30 min. Before admission, the patient had regained normal strength in all four extremities. Upon admission, the patient was still complaining of holocranial headache, which was reduced to 3 out of 10 on a NRS, nausea without vomiting, nautical dizziness, photophobia, and slight discomfort in the abdomen. There were no visual symptoms, complaints of chest pain, back pain, or cardiopulmonary symptoms.

The neurological exam was unremarkable except for slight unsteadiness in the Romberg Test, and the patient had a National Institutes of Health Stroke Scale score of 0. The patient had no cranial nerve dysfunction, did not have neck stiffness, and had a Glasgow coma scale score of 15 points. There were no clinical findings suggestive of connective tissue disease, such as a marfanoid habitus.

Upon arrival, his blood pressure was 127/56 mmHg, pulse rate 80 beats per minute, oxygen saturation 97%, and temperature 36.6 degrees Celsius. Blood tests revealed normal white blood cell counts, C-reactive protein levels, and liver and kidney parameters. Electrocardiogram demonstrated sinus rhythm with premature atrial complexes and without ST-segment deviations.

**Figure 1:**
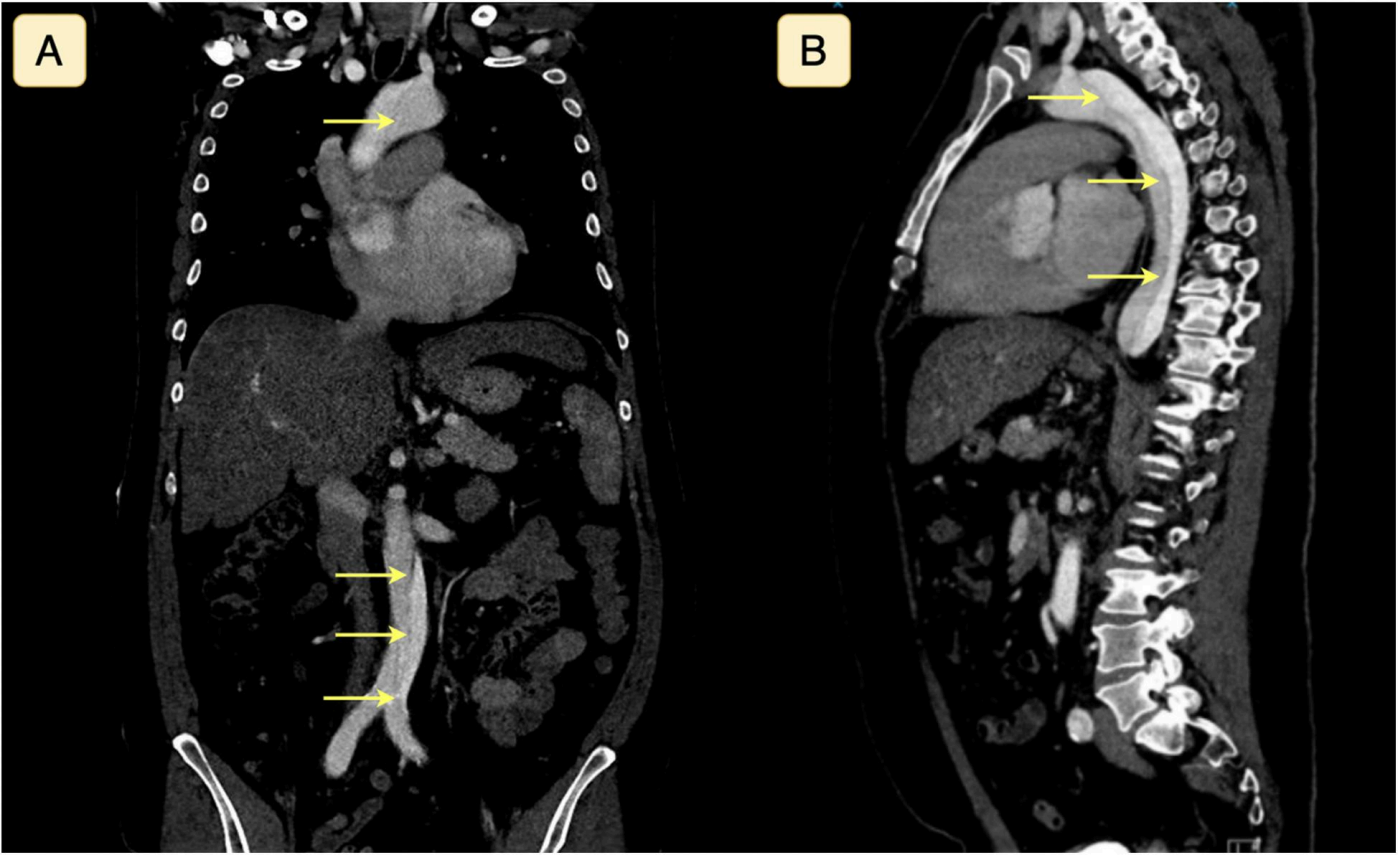
Computed tomography with angiography of the thorax and abdomen seen in coronal **(A)** and sagittal **(B)** plane, demonstrating a Stanford type A aortic dissection. The yellow arrows highlight the dissection plane separating the true and false lumina.

Non-contrast-enhanced computed tomography (CT) of the cerebrum revealed no intracranial abnormalities, including hemorrhage or ischemic lesions. Subarachnoidal hemorrhage was still suspected due to the thunderclap nature of the headache despite the normal CT scan of the cerebrum, while transitory cerebral ischemia was also considered a differential diagnosis. CT angiography of the cerebrum did not reveal any vascular abnormalities. Due to complaints of slight abdominal discomfort shortly after admission alongside the severe headache, CT angiography of the thorax and abdomen in the context of differential diagnostic purposes on the suspicion of aortic dissection was done, revealing a Stanford type A aortic dissection without concomitant affection of the carotid or vertebral arteries (Figure 1). Subsequent transthoracic echocardiography also visualized the extent of the aortic dissection from the aortic annulus to the femoral artery.

Subsequently, the patient was transferred to the department of thoracic surgery, undergoing a surgical insertion of a freestyle prosthesis at the aortic root and arch. The postoperative phase was complicated with atrial fibrillation and pericardial exudate, treated with amiodarone and subxiphoid pericardiotomy, respectively. The patient was discharged after a few weeks with follow-up in the cardiology department.

## Discussion

Aortic dissection occurs because of a breach in the intima, penetrating the intima due to high blood pressure, causing blood to enter this space with further dissection propagation (10). Hypertension and atherosclerosis are the most common predisposing factors, while Marfan's syndrome, prior cardiac surgery, and aortic aneurysm are some of the other risk factors involved in developing aortic dissection (2, 10). The incidence is approximately 3 per 100.000 each year (11).

Aortic dissections are classified based on their anatomic location and time from onset, where Stanford type A dissections involve the ascending aorta and Stanford type B dissections occur distal to the left subclavian artery without involvement of the ascending aorta (2).

**Figure 2:**
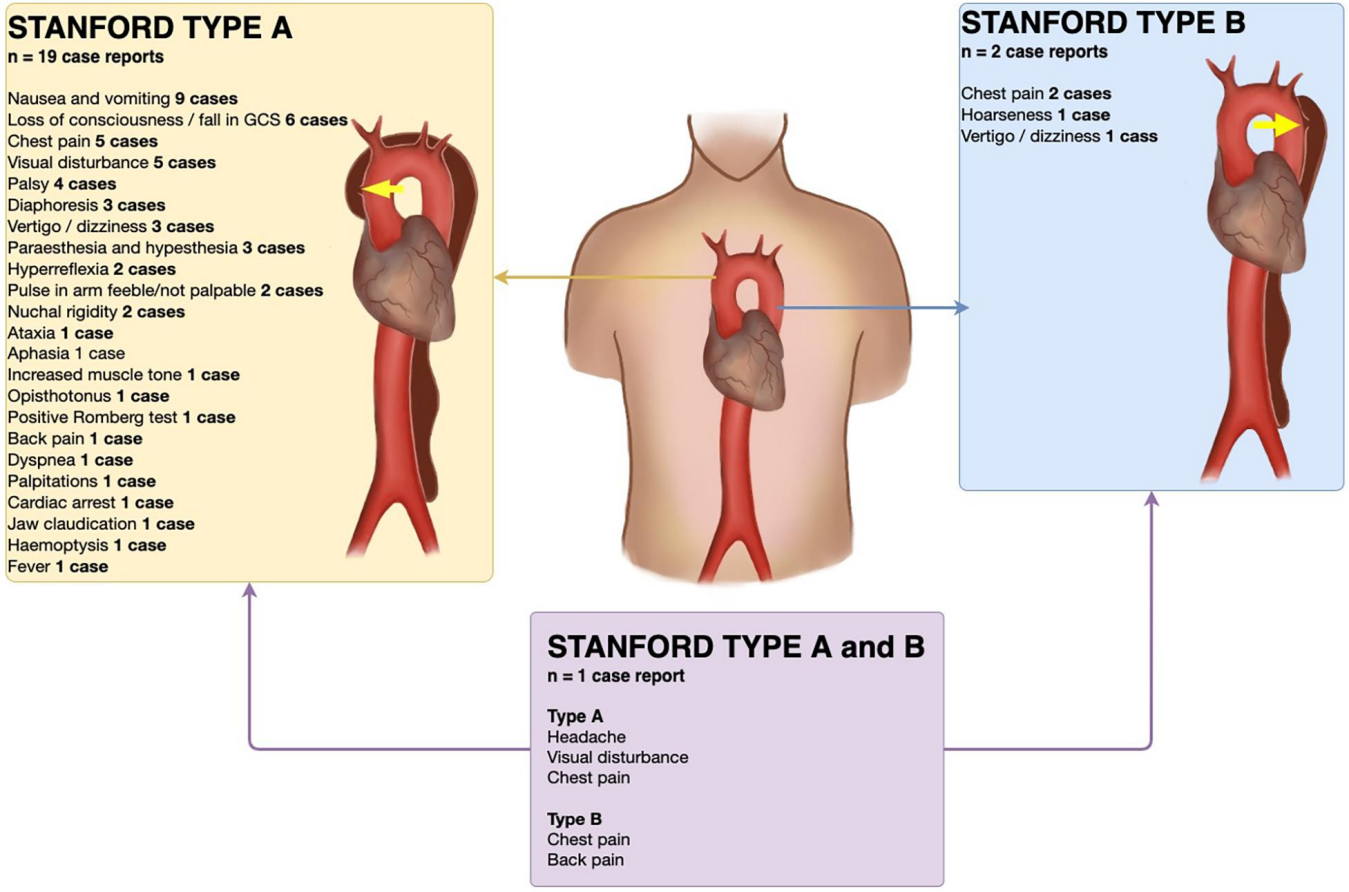
Findings and symptoms in reviewed case reports of Stanford type A and type B aortic dissections with headache as presenting symptom. Yellow arrow signifies site of dissection. Illustrated by Cecilia Ebratt. Copyright © 2025, all rights reserved.

A systematic search of the literature in the PubMed database was performed in March 2025. We aimed to include all case reports with aortic dissection with headache as a presenting symptom, where the search term “aortic dissection AND headache” was applied. 405 studies were reviewed, of which 22 case reports were included (Table 1). One study was not included due to unavailable full text. Included cases were divided into Stanford type A (*n* = 19 case reports) (1, 7, 8, 12–25), Stanford type B aortic dissection (*n* = 2 case reports) (26, 27), and one case with a patient presenting with a Stanford type A dissection progressing into a Stanford type B (Figure 2) (28). The major risk factor (*n* = 11) was hypertension, and one case reports the underlying cause as Marfan syndrome (25), one case reported paraganglioma on the right side of the aorta on the level of the celiac trunk (22), and one case reported giant cell arteritis with the focus of inflammation being the aortic wall (20). Alongside headache as the initial symptom, nausea/vomiting (*n* = 9 case reports) and loss of consciousness (*n* = 6 case reports) were the most prevalent initial symptoms. CT of the cerebrum was done in 10 studies, of which two were abnormal; one case was with dissection of extracranial arteries (17) and one case with thrombus in the right middle cerebral artery (7). Two cases of Stanford type A aortic dissections were with feeble or not palpable pulses in the brachial or radial arteries in one arm (21, 24), and 3 cases (out of 11 reporting blood pressure) reported lateralized blood pressure difference of 20–66 mmHg (12, 19, 21), with one case reporting an unrecordable blood pressure in one arm (24).

**Table 1:** An overview of case reports with aortic dissection with headache as a presenting symptom.

Author (year)	Type and location of aortic dissection after CT- angiography	Age and sex of patient	Co-morbidities	Headache debut, severity, and characterization	Other symptoms	Initial blood pressure and scanning of brain
Aoki et al. (2021) (25)	Stanford type A.	35-year-old male.	Migraine. After this case diagnosed with Marfan syndrome.	Gradual and slow onset. Worsening over 5 h. Pulsatile, and diffuse frontal throbbing pain with aura. Severity not specified.	Visual disturbances in right visual field, typical for his usual migraine.	90/48 mmHg. CT-cerebrum without hemorrhage.
Murphy et al. (2023) (24)	Stanford type A.	Male in his 40s.	No medical history.	Sudden severe right frontal headache.	Sudden sharp, intense, retrosternal chest pain radiating to the neck and lasting few seconds. No palpable right radial or brachial pulse. Diaphoresis. Loss of consciousness.	130/70 mmHg from left arm. Right arm not recordable (no pulse). CT-cerebrum without abnormalities.
Chahine et al. (2018) (23)	Stanford type A.	44-year-old female (African American).	Hypertension, Schizophrenia, and smoker (30 pack/year).	Rapidly progressive and severe (NRS 10 out of 10) throbbing frontal headache, hours before admission.	Generalized weakness. Blurry vision Dizziness.	107/51 mmHg. Day after admission uncontrolled hypertension up to 200/95 mmHg. CT-cerebrum without hemorrhage. MRI-cerebrum with punctate ischemic lesions in right middle cerebral artery territory.
Borrego et al. (2016) (22)	Stanford type A.	49-year-old female (born in Africa).	Hypertension and hyperthyroidism. After this case diagnosed with paraganglioma.	Headache, not specified further.	Chest pain. Vomiting. Haemoptysis.	200/140 mmHg. CT-cerebrum not done.
Kaeley et al. (2022) (21)	Stanford type A/DeBakey type 1.	23-year-old female.	No prior characteristics of connective tissue disorder. Otherwise not specified.	Sudden onset holocranial headache for one day. Not specified further.	Vomiting without nausea. Palpitations Diaphoresis. Right upper limb with sudden onset numbness and paraesthesia, weakness lasting two hours. The right upper extremity was cool to the touch. Hyperreflexia was found in the right upper and lower limbs with extensor plantar response was seen on the right side. Romberg's test was positive. Ataxic gait. Radial and brachial artery pulsations were feeble in the right arm.	Right arm 68/50 mmHg and left arm 134/60 mmHg. CT-cerebrum not done.
Pak et al. (2014) (20)	Stanford type A.	76-year-old female.	Hypertension. After this case diagnosed with giant cell arteritis with focus of inflammation being the aortic wall.	New onset headache 6 month prior to admission. Not specified further.	Visual field disturbance. Jaw claudication. Fever (39°C).	110–130/60–70 mmHg. CT-cerebrum not done.
Stöllberger et al. (1998) (19)	Stanford type A.	61-year-old female.	Migraine and hypertension.	Continuous bifrontal headache that was different to her known migraine and much more severe.	Right sided thoracic pain. Nausea and vomiting.	80/50 mmHg on the left arm and 105/60 mmHg on the right arm. CT-cerebrum ordered, result not reported.
Ko and Park et al. (2014) (5)	Stanford type A/DeBakey type 1.	61-year-old male.	Not specified.	Sudden thunderclap bi-frontal headache with continuous throbbing. Associated with neck pain and neck stiffness.	None.	137/70 mmHg. CT-cerebrum + angiography without abnormalities or dissection.
Singh et al. (2007) (12)	Stanford type A.	50-year-old male (Chinese).	Hypertension.	Sudden onset severe bi-frontal headache, not responding to analgesics.	Diaphoresis. 30 min after arrival drop in GCS to 14 points.	At admission 129/67 mmHg. Thirty minutes after arrival drop in blood pressure to 76/50 mmHg in the left arm and 95/60 mmHg in the right arm. CT-cerebrum without hemorrhage, infarction or mass effects.
Croft et al. (2014) (18)	Stanford type A.	43-year-old male.	Mild asthma and depression. Also taking cocaine (last used 6 weeks prior to admission).	Sudden onset headache radiating to the back of his head.	Blurred vision and flashing in the right visual field. Nausea and vomiting.	107/57 mmHg. CT-cerebrum without abnormalities.
Kamtchum Tatuene et al. (2015) (3)	Stanford type A.	44-year-old male (Caucasian).	None.	Sudden onset unilateral right-sided pressure-like headache with a progression over 30 s to NRS 10 out of 10. Two episodes of similar headache 3 and 6 days prior to admission. Pain subsided after analgesic treatment.	Right hemifacial hypoesthesia. Nausea. Mild chest pain (NRS 2 out of 10) radiating to the back and aggravated by deep inspiration.	Normal and symmetrical blood pressures. Not specified further. MRI-cerebrum without abnormalities.
Rust and Kimmig (2013) (17)	Stanford type A.	67-year-old female (Caucasian).	Diagnosed with aortic aneurism 6 months prior to admission. Otherwise, none.	Sudden onset holocranial thunderclap headache, described as of a knife-stabbing character.	Possible short-lasting motor aphasia.	Not specified. CT-cerebrum + angiography with dissection of proximal extracranial arteries.
Sharma et al. (2024) (16)	Stanford type A/DeBakey type 1.	54-year-old female.	Hypertension and hypercholesterolemia.	Severe thunderclap headache lasting for 1 h.	Vomiting for 1 h simultaneous with headache. Vertigo. Possible loss of consciousness. Slight left central facial palsy. Increased muscle tone bilaterally, greater on the left than the right. Palsy of the left sided extremities (MRC power 4 + out of 5). Hyperreflexia in the left upper extremity.	157/88 mmHg. CT-cerebrum with old ischemic lesion in the right frontal lobe and no acute lesions.
Runyan et al. (2010) (1)	Stanford type A/DeBakey type 1.	51-year-old male.	None.	Persistent headache of two months duration. Not specified further.	None.	Not specified. CT-cerebrum not done.
Nohé et al. (2005) (15)	Stanford type A	63-year-old female.	Severe arterial hypertension.	Sudden onset of excruciating headache. Not specified further.	Loss of consciousness (GCS score of 6 points).Nuchal rigidity. Opisthotonos.	160/90 mmHg in both arms. CT-cerebrum + angiography was without signs of SAH, early ischemia, or occlusion of intracerebral vessels.
Alonso et al. (2017) (7)	Stanford type A.	58-year-old male.	None.	24-h history of global headache that had become worse 4 h prior to admission.	Decreased alertness (GCS score of 13 points—E4V3M6). Palsy of left arm and face (MRC power of 3 out of 5).	145/77 mmHg. CT-cerebrum showed thrombus in the right middle cerebral artery with sign of acute ischemic infarction.
Cupa et al. (2018) (26)	Stanford type B.	63-years-old male.	Hypertension.	Discrete headaches accompanied by dizziness	Dizziness. Chest pain.	210/110 mmHg on both sides. CT-cerebrum not done.
Takeuchi et al. (2021) (14)	Stanford type A.	68-year-old woman.	Diabetes mellitus, heavy smoker, acute myeloid leukemia (subtype: M0-FAB), diagnosed 1 year before admission. During this case diagnosed with miliary tuberculosis.	Headache, not specified further.	Lumbar pain lasting for 1 week. Disorientation immediately after admission. Nuchal rigidity immediately after admission.	Not specified. CT-cerebrum not done.
Chen et al. (2005) (27)	Stanford type B.	36-year-old male.	Hypertension and gouty arthritis.	Several episodes of transient headache during a 10-day period prior to admission.	Sudden onset of hoarseness associated with headache.	111/68 mmHg. MRI-cerebrum without hemorrhage or infarction.
Mathys et al. (2004) (13)	Stanford type A.	53-year-old male.	Migraine with aura (occasional) and smoker (20 cigarettes a day over the last 3 decades).	Intense bi-frontal headache and neck pain evolving over 2 min hours before admission few minutes after onset of chest pain. Headache aggravated by head movement.	Stabbing anterior chest pain (10 min). Nausea. Dizziness. Visual disturbance with flickering eyes.	140/70 mmHg. CT-cerebrum without hemorrhage or aneurysms.
Fanelli et al. (2003) (28)	Stanford type B dissecting into a type A during endoluminal treatment.	57-year-old male.	Hypertension.	Headache, not specified further.	Stanford type B: chest and back pain. Stanford type A (during stent-graft treatment): headache, visual flashes, and mild central chest pain.	Not specified. CT-cerebrum not done.
Ramaraj et al. (2008) (8)	Stanford type A.	70-year-old male.	Seven-year history of diabetes mellitus, hypertension, hypothyroidism, and two-year history of congestive heart failure.	At admission: Left-sided headache. 3 h after admission: Headache radiating down the cervical spine.	At admission: visual disturbance with monocular blindness. 3 h after admission: Vomiting (three times). Breathlessness, Asystolic cardiac arrest leading to death.	173/88 mmHg right arm and 167/82 left arm. CT-cerebrum requested. Result not reported.

CT, computed tomography; MRI, magnetic resonance imaging; MRC, medical research council; NRS, numeric rating scale; GCS, Glascow coma scale (eyes, verbal, motor).

Patients with aortic dissections typically present with acute onset severe chest pain (72.7%) or back pain (53.2%) (2). Anterior chest pain is usually observed in patients with Stanford type A aortic dissections, while type B dissection typically presents with pain in the back and abdomen (2). In this case report, the patient complained of mild abdominal pain. However, neurological symptoms such as headache, paraplegia, transitory cerebral ischemia, or stroke are seen in about a third of patients with aortic dissection (29). Headache as the initial manifestation of aortic dissection may be due to vessel wall distension, ischemia of the pericarotid plexus, or decreased cerebral perfusion due to antegrade flow and subclavian steal (1, 17, 19). A disruption of sympathetic projections could also be a cause of pain sensations due to the affection of the ciliary ganglion and pterygopalatine ganglion by mechanical irritation of or occlusion of the vasa nervorum of the cardiac plexus and the internal carotid plexus (17).

The abovementioned neurological manifestations can divert attention away from an underlying aortic dissection (3), contributing to the substantial percentage of up to 43% of cases missed upon initial clinical evaluation (7, 9). This can ultimately have devastating consequences for the patient as treatment with r-TPA due to the suspicion of stroke has shown to have a 71% mortality rate (7, 8).

Aortic dissection can diagnosed using CT angiography, magnetic resonance imaging, and transoesophageal echocardiography (2, 12), while chest x-ray showing a widening of the mediastinum can support the diagnosis (15). Normal echocardiography or chest x-ray findings do not exclude the possibility of aortic dissection (15).

## Conclusion

This case report demonstrates a 54-year-old man presenting with thunderclap headache and focal neurological deficits as the initial manifestations of acute aortic dissection. These atypical manifestations can sometimes mimic neurological conditions, thus increasing the possibility of overlooking an aortic dissection.

Therefore, a high level of clinical suspicion for aortic dissection is warranted when patients present with a sudden severe headache accompanied by mild complaints of pain in the abdomen, chest, or back, nausea, and loss of consciousness. At the same time, asymmetry of pulse and blood pressure asymmetry of the arms should also raise suspicion. Furthermore, CT-angiography of the thorax and cerebrum is necessary to establish a timely diagnosis and subsequent treatment of aortic dissection.

## Data Availability

The datasets presented in this article are not readily available because of ethical and privacy restrictions. Requests to access the datasets should be directed to the corresponding author.
